# The management of pain: non-pharmacologic analgesia

**DOI:** 10.1186/1824-7288-41-S1-A27

**Published:** 2015-09-24

**Authors:** Patrizia Papacci, Francesca Serrao, Mikael Ghennet Tesfagabir, Velia Purcaro, Carmen Giannantonio, Costantino Romagnoli

**Affiliations:** 1 Department of Pediatrics, Division of Neonatology, Catholic University of Sacred Heart, Rome00168, Italy

## 

“Pain is an unpleasant sensory and emotional experience associated with actual or potential tissue damage, or described in terms of such damage. Each individual learns the application of the word through experiences related to injury in early life”[1]. It is now clear that premature and full-term newborns have the neuroanatomical pathways from the periphery to cortex required for nociception. In fact by the 23th week of gestation painful stimuli are associated with physiologic, hormonal, and metabolic markers of the stress response. Indeed pain perception may be greater because of immaturity of descending inhibitory pathways [2]. Preterm infants are particularly vulnerable to brief and long term effects of pain and stress because system modulating sensory experience is immature[3,4]. Neonatal intensive care involves a high number of diagnostic and therapeutic procedures which are associated with pain for preterm and sick newborn infants. In addition to immediate unpleasantness, painful experiences can imprint themselves indelibly on the nervous system amplifying and causing typically painless sensations to be experienced as pain. Pharmacological and non-pharmacological intervention (NFI) are recommended for painprevention and pain management [5]. In order to achieve optimum efficacy, both pharmacological and NFI additionally require a reduction of external stimuli, such as loud noise and bright light [6]. NFI is recommended for procedural and mild pain [7]. NFI for procedural pain is a treatment that is initiated before and during the procedure in order to reduce the physiological consequences of nociceptive transmission provoked by the procedure. Therefore NFI could be considered a “pre-emptive analgesia”. NFI activate the “gate control mechanism”, some intervention lead to an endogenous endorphin dispersal which contributes to modulation of the pain pulse at the level of spinal cord [8,9], some other may elicit activation of neuropeptides systems that can achieve an analgesic effect through the potentiation of opioid activity [10], There is sufficient evidence to support the use of NFI, particularlybreast feeding, sweet-tasting solutions, kangaroo care, non nutritive suckling, swaddling and facilitate touching for the common needle-puncture procedures [11-13]. Other NFI such as music, olfactory and multisensory stimulation areto some degree beneficial to neonates who undergo painful procedures [14,15]. Despite our limited understanding of the underlying mechanisms of actions of NFI, there seems to be few documented short-term harms from their use. NFI need a collaborative effort. Support from the administration and leadership, both formal and cultural, is crucial for the implementation of NFI[16].
